# Pregnancy and Infant Development (PRIDE)—a preliminary observational study of maternal adversity and infant development

**DOI:** 10.1186/s12887-021-02801-1

**Published:** 2021-10-15

**Authors:** Katherine Bowers, Lili Ding, Kimberly Yolton, Hong Ji, Nichole Nidey, Jerrold Meyer, Robert T. Ammerman, Judith Van Ginkel, Alonzo Folger

**Affiliations:** 1 Department of Pediatrics, University of Cincinnati College of Medicine, Cincinnati, OH USA; 2 Division of Biostatistics and Epidemiology, Cincinnati Children’s Hospital Medical Center, Cincinnati, OH USA; 3 Division of General and Community Pediatrics, Cincinnati Children’s Hospital Medical Center, Cincinnati, OH USA; 4 Division of Asthma Research, Pyrosequencing Core Lab for Epigenomic and Genomic Research, Cincinnati Children’s Hospital Medical Center, Cincinnati, OH USA; 5 Department of Anatomy, Physiology and Cell biology California National Primate Research Center School of Veterinary Medicine, University of California, Davis, CA 95616 USA; 6 Department of Psychological and Brain Sciences, University of Massachusetts Amherst, Neuroscience and Behavior Program, Amherst, MA USA; 7 Division of Behavioral Medicine and Clinical Psychology, Cincinnati Children’s Hospital Medical Center, Cincinnati, OH USA; 8 Every Child Succeeds, Cincinnati Children’s Hospital Medical Center, Cincinnati, OH USA

**Keywords:** Home visiting, Maternal adversity, Development, Longitudinal cohort

## Abstract

**Background:**

Children from socioeconomically disadvantaged families have a markedly elevated risk for impaired cognitive and social-emotional development. Children in poverty experience have a high risk for developmental delays. Poverty engenders disproportionate exposure to psychological adversity which may contribute to impaired offspring development; however the effect may be mitigated by social support and other aspects of resilience. Our objective was to determine the association between maternal stress, adversity and social support and early infant neurobehavior and child behavior at two and three years.

**Methods:**

We conducted a longitudinal mother-infant cohort study nested within a regional home visiting program in Cincinnati, Ohio. Four home study visits were completed to collect measures of maternal stress, adversity and social support and infant and child behavior. A measure of infant neurobehavior (‘high-arousal’ infant) was derived from the NICU Network Neurobehavioral Scale (NNNS) at 1 month and externalizing and internalizing symptoms were measured by the Child Behavior Checklist (CBCL) at 24 and 36 months. Linear and logistic regression identified associations between maternal risk/protective factors and infant and child behavioral measures. We used stratification and multiplicative interaction terms to examine potential interactions.

**Results:**

We enrolled n = 55 pregnant mothers and follow 53 mother–offspring dyads at 1 month, 40 dyads at 24 months and 27 dyads at 36 months. Maternal adversity and protective factors were not associated with neurobehavior at one month. However, maternal depression and measures of distress in pregnancy were significantly associated with internalizing and externalizing symptoms at 24 and 36 months.

**Conclusions:**

This pilot study established the feasibility of conducting longitudinal research within a community intervention program. In addition, although there were no statistically significant associations between maternal psychosocial factors in pregnancy and infant neurobehavior, there were several associations at 24 months, primarily internalizing symptoms, which persisted through 36 months. Future work will replicate findings within a larger study as well as explore mediators and modifiers of these associations.

## Background

Developmental delays in the pre-school and school age periods, which affect as many as 13% of toddlers and are increasing in prevalence, have a demonstrated effect on long-term physical and mental health and well-being [1]. Sociodemographic disparities are associated with an increased risk for developmental impairment [2]. For example, poverty carries disproportionate risks for childhood developmental delays [3], impaired language and literacy [4], and negative social-emotional function [5, 6]. In fact, children in poverty are 40% more likely to experience developmental delays relative to children not in poverty. Poverty also engenders disproportionate exposure to adversity including parental/child psychosocial stressors (e.g., violence, relocation, and food insecurity) and psychological distress (e.g., maternal depression). A broad literature has identified an association between maternal psychological stress and adversity and developmental outcomes. This association may explain the disproportion of poor developmental outcomes among families with high sociodemographic risk [7, 8]. Despite a growing body of literature, the types and timing of stressors, modifiers of the association, and the underlying biologic mechanisms remain uncertain.

A leading hypothesis linking maternal social factors to offspring development is through epigenetic alterations in utero [9, 10]. Developmental programming, or the fetus’s physiologic adaptations to characteristics of the intrauterine environment, is thought to be described by epigenetic processes and is increasingly recognized as a contributing factor to impaired development [11]. The most highly studied mechanism of programming is DNA methylation, which is the process by which methyl groups bound to CpG dinucleotides affect the level of genetic transcription. While gestation represents an important window for developmental programming, the extent of programming may depend on adverse events that occur long before conception [12–14]. In addition, programming effects may be modified by the postnatal environment, including positive experiences such as social support. DNA methylation analyses were conducted, but are not described in this paper.

The overall goal of our research is to reduce developmental health disparities by optimizing home visiting practices which serve at-risk families. The research goal of The PRegnancy and Infant DEvelopment (PRIDE) Study was to establish the feasibility of assembling an observational, longitudinal cohort study within the framework of an ongoing home visiting intervention and then to understand the intergenerational impact of maternal stress, adversity, and social support on early infant neurobehavior and child development. This paper will describe the overall study design and preliminary associations between stress, adversity, social support, and infant and child behavior.

## Methods

### Overall study procedures

**Fig. 1:**
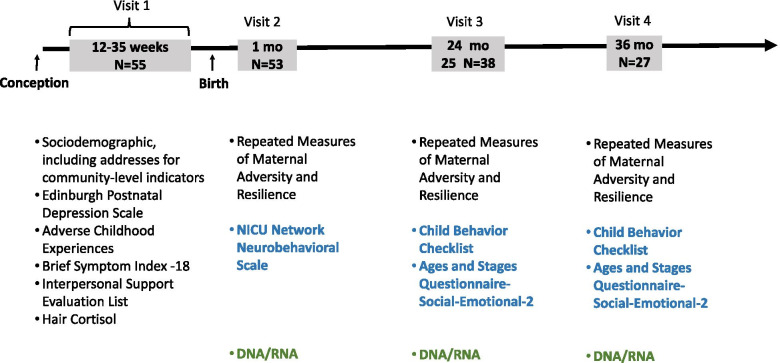
Overview of PRIDE-Cincy study visits and data collection

The PRIDE Study is a mother-infant cohort based in Cincinnati, Ohio. The pilot wave of the study involved four home study visits; the first visit occurred during the second or third trimester of pregnancy, the second visit occurred at 3–5 week postnatal, the third visit at 24 months and the fourth visit at 36 months (Fig. 1). The purpose of the first study visit was to obtain informed consent, collect data on maternal stress, adversity, and social support during childhood and pregnancy, and collect a hair sample. During the second visit, we assessed infant neurobehavior and collected buccal cells from the infants for DNA methylation analysis. The third and fourth visit continued to collect maternal adversity and protective factors while collecting a buccal sample from children along with child behavior. A small monetary incentive was provided to participants at each study visit. For visits one and two, the incentive was $20 and $30, respectively, while visits three and four provided a $60 incentive on card that functions like a debit card. This research was approved by the Institutional Review Board of Cincinnati Children’s Hospital Medical Center.

### Population and recruitment

We enrolled 55 mother-infant dyads who were participating in Every Child Succeeds (ECS), a home visiting program which serves the Greater Cincinnati area, including Southwest Ohio and Northern Kentucky, by providing evidence-based services to first-time, at-risk mothers from pregnancy until the child is age 3 years. Enrollment for PRIDE was exclusively from Hamilton County, Ohio. Approximately 25% of eligible mothers in the region participate in the ECS program. Women can be self-referred or referred by a provider. Women who enroll prenatally receive weekly, bi-weekly, or monthly home visits depending on the gestational week. Postnatal visits occur with similar frequency and include regular developmental screening using the Ages and Stages Questionnaire (ASQ)-III beginning at age four months. The ASQ-III screens children through age five for developmental delays and disabilities.

ECS home visitors referred all eligible pregnant participants to our PRIDE study team, who contact each woman to confirm eligibility and schedule the first study visit. In addition to participants in ECS, eligibility criteria for PRIDE included pregnancy prior to 36 weeks,18 years of age or older, and English speaking.

### Maternal stress and adversity measures

Several measures of maternal adversity and stress were collected at each study visit. The Adverse Childhood Experiences Scale (ACE) [15], a 10-question self-report measure, captures abuse, neglect, and household dysfunction through age 18. The Edinburgh Postnatal Depression Scale (EPDS) [16] is a 10-item self-report measure of depressive symptoms and is validated for use prenatally [17]. It collects depressive symptoms that occurred over the past week based on a four-point scale indicating frequency and severity. The Brief Symptom Index-18 (BSI-18) is a brief measure used to screen for common psychiatric disorders including depression, anxiety, and somatization [18]. The Pregnancy Experience Scale (PES) Brief version measures pregnancy-specific contributors to psychological state using the top 10 items from the original scale with comparable validity and reliability [19, 20]. The Perceived Stress Scale (PSS), the most widely used instrument to measure perceived stress [21], was designed for community samples and is easily interpreted. With the exception of the ACE scale and PES, adversity measures were collected again at visits two, three and four.

Neighborhood-level adversity was determined by linking birth address data with socioeconomic variables from the American Community Survey from the U.S. Census Bureau. Variables included the percent of households in the neighborhood with assisted income, percent with a high school education, the mean income, percent with no health insurance, the percent who experience poverty and the percent of vacant housing. In addition, we included a deprivation index which is based on a principal components’ analysis of these six measures [22, 23]. The deprivation index ranges from zero to one.

### Biologic measure of stress

For an objective measure of stress, we measured cortisol accumulation in hair. Rather than a snapshot of cortisol at a single time point, as when measured in saliva, hair cortisol represents an accumulation of cortisol. One centimeter of hair represents approximately one month of cortisol accumulation, We collected hair from 30 women. Of the 25 women without a hair sample, a majority were willing but were wearing a wig or weave and therefore unable to provide natural hair. Hair was cut from the occipital vertex using a standard protocol. Our laboratory methods for measuring hair cortisol included duplicate analyses and rigorous quality control standards and are described in detail previously [24]. Briefly, hair is weighed on an analytical balance scale and washed with isopropanol to remove contamination on the external part of the hair. The isopropanol is then dried and the sample is ground to a fine powder. Cortisol is measured using a commercial enzyme immunoassay (Salimetrics) and converted to pg per mg of hair. To determine hair cortisol concentrations the assay readout is converted to pg cortisol per mg sample weight. An limit of detection (LOD) taking into account sample weight was calculated for each individual hair sample that, when reconstituted and analyzed, yielded a cortisol value below the overall assay LOD. There were three samples below the weight adjusted LOD. Intra- and inter-assay coefficients of variation for this assay are both < 10%.

### Maternal Social Support

The Interpersonal Support Evaluation List (ISEL) [25], a widely used measure of social support, measures 40 items regarding the availability of tangible and emotional support. Scores on four subscales are derived: Appraisal, Belonging, Self-Esteem, and Tangible. The Appraisal scale measures whether individuals have the ability to talk to someone about problems. The Belonging scale measures whether there are people to do things with. The Self-Esteem scale measures whether one has a positive comparison of their self to others and the Tangible scale measures whether there is material aid available. The IESL was collected at each visit.

### Infant and child development

Infant neurobehavior was measured using the NICU Network Neurobehavioral Scale (NNNS) at the second study visit [3–5 weeks postnatal] [26]. The NNNS measures three components of neurobehavior including: 1) CNS integrity and neurological functions, such as active and passive tone and primitive reflexes; 2) infant behavior to assess neurologic states as well as sensory and interactive responses; 3) signs of stress which can manifest as overt or subtle signals during the course of the examination. The exam was developed based on previous validated infant examinations, in particular the Neonatal Behavioral Assessment Scale (NBAS) [27]; however, a major difference is the NNNS incorporates a standardized administrative format developed to minimize the effect of the examiner on the assessment [26]. While the exam was developed for high-risk infants, it is appropriate for all infants regardless of risk for neurobehavioral deficits [26]. There are 114 individual test items. The approximately 30 min exams were completed by a trained examiner who is also a certified trainer on the NNNS (KY).

Summary scores (domains) were developed using a combined conceptual and statistical approach to aggregate scores from the individual NNNS items to describe 13 dimensions of neurobehavior including: habituation, attention, arousal, self-regulation, special handling required to acquire orientation items, movement quality, excitability, lethargy, non-optimal reflexes, asymmetrical reflexes, hypertonicity, hypotonicity and signs of stress. For all subscales, higher scores reflect a greater tendency toward that dimension regardless of whether it is a positive or negative trait. In addition to evaluating individual dimensions of neurobehavior, we employed previously identified profiles of behavior developed within an independent Cincinnati cohort [28]. Latent profile analyses classified the 13 dimensions to identify infants with profiles described as ‘high-arousal’, ‘hypotonic’ and ‘social’ [28]. Our primary outcome variable was having a ‘high-arousal’ infant and ‘social’ was the reference (no infants were identified as ‘hypotonic’ in this sample).

At visits three and four [24 and 36 months], a Child Behavior Checklist (CBCL) was collected. The Child Behavior Checklist (CBCL/1½-5) is a parent-report questionnaire that will be used to measure behavior and emotional functioning including externalizing and internalizing behaviors [29].

### Statistical analyses

All variables were examined for errors, inconsistencies, incomplete information and distributional properties. Psychometric assessments were scored based on guidance from the test publishers. Demographics were summarized using means (standard deviations) for continuous variables and number (percent) for categorical variables. A cortisol measure was available for 29 of the 30 hair samples, and one sample outlier was excluded, resulting in 28 samples available for analyses.

To control for potential confounding variables, we employed logistic regression analyses to determine the odds of having a high-arousal infant. Potential covariates included maternal age (years), race (black versus white/Asian/multi-race), and maternal ACEs (< 2, ≥ 2). Spearman correlation coefficients estimated the correlationbetween maternal adversity and internalizing and externalizing symptoms, as well as the CBCL total score.

## Results

We recruited from a limited sample of 8 home-visiting agencies in Hamilton County. Over 6 months, we received 63 eligible referrals, of which only 7 women refused participation (89.9% participation). Of the 56 women interested in participating, 55 prenatal visits were completed. Fifty-three postnatal visits were completed (2 participants lost to follow-up; 84.1% participation).

The mean age of women participating in The PRIDE Study was 21.8 years, a majority were black/African American (61.2%), and few were Hispanic (5.5%) (Table 1). A low percentage of mothers (5.6%) and slightly higher percentage of fathers (15.1%) had less than a high school education. While only 12.7% were unemployed, one quarter of the women had an annual household income less than $15,000.

**Table 1:** Sociodemographic characteristics and adversity measures of Mothers in the Pregnancy and Infant Development Study expressed as mean (standard deviation) for continuous variables and percentages for categorial variables

**Individual Sociodemographic Indicators**	Baseline
**n**	53
Age, mean ± sd	21.8 (3.3)
Black, Non-Hispanic,%	61.2
Hispanic,%	5.5
< High school,%	5.6
Father < high school,%	8 (15.1)
Unemployed,%	12.7
Annual Income < $15 k,%	24.5
**Adversity and Protective Measures**	
ACEs	2.1 (1.8)
≥ 2ACEs, %	34.0
PSS4	5.7 (3.9)
PSS10	16.4 (8.4)
PES hassles	22.2 (5.9)
PES uplifts	13.8 (6.7)
PES frequency of hassles	9.2 (1.5)
PES frequency of uplifts	7.0 (2.3)
Ratio frequency hassles: uplifts	1.7 (1.4)
Ratio intensity hassles: uplifts	1.4 (0.6)
Cortisol ^a^	6.1 (4.6)
PES/PSS combined	
Low	2 (3.8)
Mid	47 (88.7)
High	4 (7.6)
Social Support	
Appraisal	23.4 (5.4)
Belonging	22.1 (6.9)
Self-esteem	22.0 (4.6)
Tangible	22.1 (6.5)
^a^n = 28	
**Community Level Sociodemographic Indicators**	
Assisted income, %	0.26 (0.14)
High school education, %	0.84 (0.08)
Median income, mean $	38,287 (16,700)
No health insurance, %	0.14 (0.05)
Poverty, %	0.29 (0.16)
Vacant housing, %	0.16 (0.11)
Deprivation index	0.48 (0.13)
Distance to major roadways, miles	2,976 (3,370)

^a^*ACE* Adverse Childhood Experiences, *PSS* Perceived Stress Scale, *PES* Pregnancy Experiences Scale

Table 2 presents the association between adversity measures in pregnancy and having a ‘high-arousal’ infant (primary infant outcome) adjusting for potential confounders. There was no statistically significant association between the ratio of the frequency of hassles and a ‘high-arousal’ infant (odds ratio (OR) = 1.47, 95% confidence interval (CI): 0.93, 2.33) adjusting for maternal age, race and early adversity (ACEs). There was also no association between the ratio of the intensity of hassles to uplifts (OR = 1.92, 95%:0.53, 6.99). Cortisol accumulation in pregnancy was not associated with having a ‘high-arousal’ infant.

**Table 2:** Association between adversity, stress, and social support in pregnancy and having a ‘high-arousal’ infant compared with having a ‘social’ infant

	Unadjusted OR (95% CI)	Adjusted OR (95% CI)
**Maternal age**	1.18 (0.97, 1.43)	1.28 (1.02, 1.59)
**ACEs**	0.91 (0.70, 1.19)	0.86 (0.62, 1.18)
≤ 2	1.00 (ref)	1.00 (ref)
> 2	1.25 (0.34, 4.66)	0.78 (0.17, 3.56)
**Race**		
Other	1.00 (ref)	1.00 (ref)
Black	0.28 (0.07, 1.03)	0.18 (0.04, 0.83)
**Perceived Stress **^**a**^	0.95 (0.89, 1.04)	0.95 (0.86, 1.05)
**Pregnancy-Specific Stress **^**a**^		
Frequency hassles: uplifts	1.22 (0.82, 1.83)	1.47 (0.93, 2.33)
Intensity hassles: uplifts	1.88 (0.64, 5.47)	1.92 (0.53, 6.99)
**Cortisol (n = 28) **^**a**^		
pg./mg	0.86 (0.66, 1.12)	0.65 (0.80, 1.19)
≥ 5.5 versus < 5.5	0.60 (0.11, 3.30)	0.67 (0.10, 4.25)
**Social Support (highest Quartile versus Quartiles 1–3) **^**a**^		
Appraisal	1.56 (0.38, 6.41)	1.31 (0.26, 6.69)
Belonging	4.90 (0.92, 25.9)	6.38 (0.76, 54.48)
Self-esteem	1.05 (0.23, 4.76)	1.36 (0.22, 8.47)
Tangible	2.76 (0.61, 12.47)	4.0 (0.64, 25.0)

*OR* Odds Ratio, *CI* Confidence Interval, *ACE* Adverse Childhood Experiences, *ref* references

^a^Adjusted Odds Ratio –Models adjusted for ACE (≤ 2/ > 2), maternal age, race (black/other)

Correlations between maternal adversity in pregnancy and child behavioral problems at 24 and 36 months are displayed in Table 3. Several factors including psychological distress, depression, perceived stress and a measure of pregnancy experiences were statistically significantly associated with internalizing symptoms at 24 months. These factors, in addition to adverse childhood experiences, were also associated with externalizing symptoms. While statistical significance did not remain for all factors, the effect persisted through 36 months for most associations that were evident at 24 months.

**Table 3:** Correlation between maternal adversity in pregnancy and internalizing symptoms, externalizing symptoms and the Child Behavior Checklist (CBCL) total score at 24 and 36 months

		24 months (*n* = 40)		36 months (*n* = 27)	
**CBCL**	**Maternal Adversity**	Coefficient	P value	Coefficient	*P* value
**Internalizing symptoms**	Adverse Childhood Experiences	0.72	0.25	0.50	0.45
	Psychological Distress (BSI-18)	0.31	0.03	0.39	0.01
	Depression	0.51	0.03	0.86	0.002
	Perceived Stress	0.38	0.04	0.57	0.01
	Ratio Frequency hassles:uplifts	-1.46	0.21	-0.2	0.87
	Ratio Intensity hassles:uplifts	-6.98	0.02	-1.40	0.75
**Externalizing symptoms**	Adverse Childhood Experiences	1.696	0.01	0.81	0.24
	Psychological Distress (BSI-18)	0.494	< 0.001	0.41	0.017
	Depression	0.731	0.002	0.40	0.22
	Perceived Stress	0.598	0.002	0.29	0.25
	Ratio Frequency hassles:uplifts	-1.463	0.21	-0.20	0.87
	Ratio Intensity hassles:uplifts	-6.979	0.02	-1.40	0.75
**Total score**	Adverse Childhood Experiences	1.282	0.04	0.80	0.25
	Psychological Distress (BSI-18)	0.424	0.002	0.43	0.01
	Depression	0.683	0.003	0.69	0.03
	Perceived Stress	0.519	0.01	0.46	0.07
	Ratio Frequency hassles:uplifts	-1.913	0.09	-0.61	0.63
	Ratio Intensity hassles:uplifts	-8.467	0.004	-5.45	0.20

## Discussion

In our pilot study of 53 mother-infant pairs, few statistically significant associations were identified between adversity and protective factors in pregnancy and infant neurobehavior. However, several factors including maternal depression, perceived stress, and overall distress were associated with child internalizing and externalizing behaviors and 24 and 36 months. Although statistically significant associations with neonatal measures were not observed, several effect estimates were observed in the hypothesized direction and a few associations approached significance. For example, pregnancy-related stress, including the ratio of the frequency and intensity of hassles to uplifts, were associated with 1.5 and 2.0 times the odds of having a high-arousal infant, respectively (*p* > 0.05). Several other non-statistically significant associations presented important study questions for a larger cohort and will be discussed.

Limitations should be mentioned. At the 24- and 36-month follow-up visits we were able to re-enroll 75% and 51% of participants, respectively. If those who were unable to be contacted differed from the enrolled participants by levels of adversity and infant development, our results may be biased. A key feature of the larger longitudinal study will be improving retention throughout the planned study period. Our pilot study also had important strengths including multiple measures of adversity and longitudinal study design. Many of our results are generally in line with previous studies and confirm the conclusions of a recent critical review of the literature [30]. However, we also observed associations that did not confirm our original hypotheses. For example, measures of social support were not inversely associated with infant neurobehavior. In fact, some measures of social support were observed to be higher among those mothers who had a ‘high-arousal’ infant and those who showed ‘signs of stress’ (data not shown). There are a couple of explanations for these findings. First, our pilot sample size may have been too small to identify subtle effects of social support, and any associations we did observe could have been due to random fluctuations. In addition, social support is unlikely to impart its effects in isolation. Rather, it may be the combined effects of social support, stress and adversity that is most relevant to offspring development. Understanding the interactions between these measures will be a major objective of a large-scale observational study of a similar design that we are currently developing. Associations may also vary by other factors such as race. For example, the association between cortisol and infant stress was higher among non-black women (OR = 4.80, 95% CI: 0.40, 58.0) versus OR = 1.60 (0.10, 24.7) for black women (data not shown). Evaluating interactions was beyond the scope of this pilot study as identifying statistically significant interactions requires a large sample size. However, future work will determine whether associations truly vary by race, why any heterogeneity exists, and what the implications are for mothers and their infants.

We evaluated infant neurobehavior at 3–5 weeks for several reasons. First, we wanted a measure that was proximal to our exposure assessments. There are a multitude of factors that affect development in the post-natal environment, and we wanted to reduce the impact of these factors, therefore increasing the opportunity to observe associations with pregnancy stress and adversity. In addition, the NNNS is a comprehensive and direct measure of infant neurobehavior that was developed for research purposes. Prior studies identified statistically significant associations between NNNS and developmental outcomes measured at one, three [28] and 4.5 years [31]. Determining the association between newborn neurobehavior and the developmental trajectory in early childhood will be an objective of future work.

## Future research

The next step for the pilot study is to evaluate the role of DNA methylation of several stress-response genes in the association between maternal adversity and infant and early childhood neurobehavior. Buccal cells DNA was collected at each of the three postnatal visits. Our overall research goal is to improve developmental outcomes, specifically among families of high sociodemographic risk and those participating in home visiting. Since our study is nested within a home visiting program, we are well positioned to tailor services to maximize effectiveness based on findings from this proposed work. However, it is necessary to further elucidate the complex pathways we have begun to uncover. Given the complexity of the causal framework, a major focus will be identifying mediators and modifiers of the association between adversity and infant development.

## Conclusions

Our pilot study, PRIDE, established the feasibility of conducting observational cohort studies within the framework of an ongoing home visiting program. In addition, we identified several interesting preliminary findings that will be followed up in a larger cohort study.

## Data Availability

The datasets used and/or analyzed during the current study are available from the corresponding author on reasonable request.

## Abbreviations

OR: Odds ratio
CI: Confidence interval
NNNS: NICU Network Neurobehavioral Scale
